# The role and therapeutic potential of SIRTs in sepsis

**DOI:** 10.3389/fimmu.2024.1394925

**Published:** 2024-04-16

**Authors:** Jiaqi You, Yilin Li, Wei Chong

**Affiliations:** ^1^ Department of Emergency, The First Hospital of China Medical University, Shenyang, China; ^2^ Department of Thoracic Surgery, The First Hospital of China Medical University, Shenyang, China

**Keywords:** SIRTs, sepsis, inflammatory response, MODS, medication

## Abstract

Sepsis is a life-threatening organ dysfunction caused by the host’s dysfunctional response to infection. Abnormal activation of the immune system and disturbance of energy metabolism play a key role in the development of sepsis. In recent years, the Sirtuins (SIRTs) family has been found to play an important role in the pathogenesis of sepsis. SIRTs, as a class of histone deacetylases (HDACs), are widely involved in cellular inflammation regulation, energy metabolism and oxidative stress. The effects of SIRTs on immune cells are mainly reflected in the regulation of inflammatory pathways. This regulation helps balance the inflammatory response and may lessen cell damage and organ dysfunction in sepsis. In terms of energy metabolism, SIRTs can play a role in immunophenotypic transformation by regulating cell metabolism, improve mitochondrial function, increase energy production, and maintain cell energy balance. SIRTs also regulate the production of reactive oxygen species (ROS), protecting cells from oxidative stress damage by activating antioxidant defense pathways and maintaining a balance between oxidants and reducing agents. Current studies have shown that several potential drugs, such as Resveratrol and melatonin, can enhance the activity of SIRT. It can help to reduce inflammatory response, improve energy metabolism and reduce oxidative stress, showing potential clinical application prospects for the treatment of sepsis. This review focuses on the regulation of SIRT on inflammatory response, energy metabolism and oxidative stress of immune cells, as well as its important influence on multiple organ dysfunction in sepsis, and discusses and summarizes the effects of related drugs and compounds on reducing multiple organ damage in sepsis through the pathway involving SIRTs. SIRTs may become a new target for the treatment of sepsis and its resulting organ dysfunction, providing new ideas and possibilities for the treatment of this life-threatening disease.

## 1 Introduction

### 1.1 Background

Sepsis is a life-threatening organ dysfunction arising from a dysregulated host response to infection (1). Among hospitalized patients with or suspected of having an infection, organ dysfunction severity can be categorized using the Sequential Organ Failure Assessment (SOFA) score, with a score of two or higher prompting a sepsis diagnosis (2). According to the Global Burden of Disease Study, in 2017, there were approximately 49 million new cases of sepsis worldwide, contributing to 11 million sepsis-related deaths, which accounted for nearly 19.7% of all global deaths (3).

At the onset of sepsis, primitive immune cells detect pathogen-associated molecular patterns (PAMPs) released by infectious agents via pattern recognition receptors, like Toll-like receptors (TLRs) (4, 5). This interaction triggers an inflammatory cascade via the activation of canonical pathways, such as NF-κB (6). When an overwhelming inflammatory response ensues, a surge in pro-inflammatory cytokines (TNF-α, IL-1β, IL-6, IL-8) occurs, alongside activation of the complement system and further release of inflammatory mediators like C3 and C5 (7, 8). Excessive inflammation and immune suppression may persist or occur simultaneously (9). During immune suppression, an abundance of anti-inflammatory cytokines like IL-4 and IL-10 is produced, leading to extensive depletion of immune effector cells through apoptosis and dysfunction, and an overproduction of immune regulatory cells (10). This compromises the body’s immune defenses, heightening the risk for secondary infections by opportunistic pathogens and viruses. In such cases, patients are confronted with a grimmer prognosis and elevated mortality risk (11). During sepsis, the body’s metabolic state undergoes significant changes. On one hand, metabolic activities accelerate to meet the increased demand for energy and biosynthetic precursors by immune cells, as evidenced by the enhanced rate of glycolysis. On the other hand, sepsis can lead to abnormalities in metabolic pathways, such as mitochondrial dysfunction, affecting the generation and utilization of energy. This metabolic disarray not only weakens the cellular response to infection but can also result in damage or even death of cells and tissue organs, releasing damage-associated molecular patterns (DAMPs) (12, 13). Concurrently, platelet and neutrophil activation, endothelial cell injury, and coagulation pathway perturbations foster the formation of microcirculatory immune thrombi (14–16), resulting in maldistribution of blood flow, dysoxia, and exacerbation of organ injury (17), culminating in multiple organ dysfunction syndrome (MODS). The excessive production of reactive oxygen species (ROS) induces oxidative stress, damaging cell membranes, proteins, and DNA, leading to cellular dysfunction and even death. Furthermore, oxidative stress can activate various inflammatory pathways, promoting the release of inflammatory factors, exacerbating systemic inflammatory responses, and creating a vicious cycle.

Presently, consensus on treatment strategies is limited to a few key points, such as fluid resuscitation, hemodynamic management, antimicrobial therapy, and organ support and protection (18). Although advancements in diagnostic and treatment strategies have improved survival rates for sepsis patients, more effective methods are required to enhance long-term prognoses and reduce the incidence of sequelae. Recent research endeavors increasingly seek to intervene at pivotal junctures within the pathophysiological mechanisms of sepsis, with the aim of obstructing or mitigating the progression of sepsis-induced MODS. However, no effective molecular-targeted therapies have been established to date. Since 1982, scientists have conducted extensive preclinical research on molecular-targeted therapies for sepsis, including corticosteroids, TLR4 receptor antagonists, anti-TNF-α agents, and interventions targeting cytotoxic effects and the coagulation cascade. Yet, phase III clinical trials for these approaches have failed to demonstrate evidence of improved long-term survival rates (19).

### 1.2 Sirtuin family

Chromatin consists of DNA and histones, where the lysine residues of histones possess positive charges at neutral pH, facilitating a tight association with the negatively charged DNA. The acetylation of histones neutralizes their positive charges, favoring dissociation from DNA. This alteration enables the specific binding of various transcription factors and co-activators to DNA binding sites, activating gene transcription. The level of histone acetylation is co-regulated by Histone Acetyltransferases (HAT) and Histone Deacetylases (HDAC) (20), subsequently influencing the flexibility of the chromatin structure. This regulation plays a pivotal role in the control of gene expression and the activity of metabolic enzymes, extensively affecting cellular physiological processes, metabolic levels, and cellular functions (21, 22). Moreover, through altering biological behaviors, it changes the state of diseases in numerous pathophysiological processes (23, 24).

Histone deacetylases (HDACs) can be categorized into two principal classes based on their catalytic mechanisms: the eleven classical zinc-dependent HDACs and the seven NAD+-dependent Sirtuins (SIRTs). Comprised of approximately 275 amino acids, these enzymes possess a highly conserved catalytic core domain. Certain SIRT proteins also feature unique N-terminal and C-terminal extension regions that confer specialized functions (25, 26). These regions have been found to participate in a variety of roles, including cellular localization, protein-protein interactions, regulation of intrinsic enzymatic activity, and modulation of protein oligomerization states (27). They catalyze the deacetylation of both histone and non-histone substrates, and their specificity for different substrates is dictated by the biological processes they are involved in (28). Regarding cellular localization, SIRT1, SIRT6, and SIRT7 are predominantly nuclear, with SIRT1 shuttling to the cytoplasm under certain conditions. SIRT6 mainly resides within chromatin, regulating genes associated with metabolism, and NF-κB-dependent inflammatory genes (29). SIRT7 plays a crucial role in maintaining genomic integrity and stability within the nucleus (30, 31). SIRT2 is primarily located in the cytoplasm; however, studies have also demonstrated its presence in the nucleus during mitosis (32). SIRT3 and SIRT5 are mainly localized in the mitochondria, where they regulate mitochondrial energy metabolism. In addition to their deacetylation function, due to its unique structural characteristics, SIRT5 also possesses desuccinylation and demalonylation enzyme activities. Moreover, SIRT4 and SIRT6 exhibit ADP-ribosyl transferase activity, with SIRT6 also possesses strong long-chain fatty acylase enzyme activity (33–36). Additionally, it has been shown that SIRT1-3 also have the function of regulating the level of purified histones lactylation (37), which is similar to histone acetylation, is involved in modulating the accessibility and activity of genes by regulating chromatin structure and gene expression, it affects a wide range of biological processes, including cell growth, differentiation, response to environmental signals, and disease occurrence (38, 39).

This review focuses on the SIRTs, emphasizing their role and mechanisms within the pathophysiological processes of sepsis, particularly their specific functions in the context of MODS caused by sepsis. Furthermore, we discuss the potential of SIRTs as target candidates for the treatment of MODS in sepsis.

## 2 SIRTs and pathophysiology of sepsis

The essence of sepsis lies in the imbalance between pro-inflammatory and anti-inflammatory responses, a critical factor leading to patient mortality. During the two stages of sepsis, the Sirtuin family is involved in regulating a variety of signaling-related cellular processes. Evidence suggests that during the early pro-inflammatory stage, the levels of Sirtuin 1, 2, 3, and 6 decrease (40, 41), while the acetylation levels of the NF-κB p65 subunit increase, enhancing transcriptional activity and initiating an inflammatory cascade that releases a large number of pro-inflammatory mediators (41–45). Subsequently, the excessive inflammatory response can further damage organ function. Due to the extensive consumption of immune cells and the body’s self-protective actions toward various organs, an anti-inflammatory state is presented. The anti-inflammatory properties of SIRT1, 2, and 6 are primarily achieved through mediating the deacetylation of the transcription factor NF-κB p65 subunit, reducing its nuclear accumulation, and decreasing its affinity for binding to the promoter DNA of target genes. This interaction affects p65’s interaction with co-activators, ultimately reducing the transcriptional activity of NF-κB and lowering the expression of NF-κB-dependent pro-inflammatory cytokines/chemokines and adhesion molecules in immune cells and endothelial cells, thereby achieving an anti-inflammatory effect (46–48). Conversely, SIRT5 reduces the interaction between SIRT2 and NF-κB p65, effectively increasing the acetylation level and thereby enhancing the pro-inflammatory response (49). In addition to regulating the inflammatory process, SIRTs play an important role in cellular energy metabolism and oxidative stress. They interact and synergize to regulate cellular energy metabolism by modulating AMPK, PGC-1α, and FOXO signaling pathways. They activate AMPK, promoting ATP production and maintaining metabolic balance. SIRT1 also activates PGC-1α, enhancing mitochondrial function and biogenesis. Additionally, SIRTs suppress oxidative stress by inhibiting ROS production and modulating antioxidant enzymes, reducing cellular damage. It controls ROS-generating enzymes like NOX and regulates antioxidant enzymes such as SOD and GPx to enhance the cell’s antioxidant capacity. Thus, during different stages of sepsis, members of the Sirtuin family interact with each other to precisely regulate the progression of sepsis. We will illustrate the significant role of the SIRTs in the development and progression of sepsis from the perspectives of their involvement in the specific components of the sepsis immune response and their role in regulating energy metabolism and oxidative stress damage during sepsis.

### 2.1 SIRTs and cell immune response in sepsis

In sepsis, the host’s innate immune system represents the first line of defense and includes a variety of immune cells such as neutrophils, monocytes/macrophages, dendritic cells, they activate the adaptive immune system, such as T cells, and non-immune cells such as endothelial cells. The cells play crucial roles in MODS associated with sepsis. The Sirtuin family can regulate the activation or differentiation of immune cells within the immune system, playing a modulatory role in the pro-inflammatory/anti-inflammatory responses characteristic of sepsis. Focusing on the research into the Sirtuin family’s involvement in immune/non-immune cell metabolism and inflammatory responses promises a new direction for mitigating MODS in sepsis.

Monocytes-macrophages are ubiquitously distributed throughout various tissues and play a pivotal role during the pro-inflammatory phase of sepsis by secreting vast amounts of pro-inflammatory cytokines and chemokines, exacerbating the inflammatory response (50, 51). In the anti-inflammatory stage of sepsis, excessive apoptosis of macrophages leads to immune dysfunction and organ damage (52). Once stimulated appropriately, macrophages can polarize into two phenotypes: M1 macrophages, which primarily engage in anti-infection and pro-inflammatory reactions; and M2 macrophages, which play significant roles in suppressing inflammation and facilitating tissue repair (53). SIRT1 is capable of regulating monocyte functions through NF-κB and the peroxisome proliferator-activated receptor γ coactivator 1 (PGC-1), promoting the expression and activation of SIRT1 in macrophages, leading to a downregulation of M1 polarization markers such as TLR4, p-NF-κB, IL-1β, and iNOS, while upregulating M2 polarization markers like Arg1, thus facilitating the polarization towards M2 macrophages and mitigating the inflammatory response (54, 55). In addition to influencing macrophage immune phenotypes, Sirt1 binds to deacetylated lysine residues (28-30) of HMGB1, forming a stable nuclear complex and inhibiting its release. During sepsis, macrophages uptake lactate via MCTs, facilitating HMGB1 lactylation through p300/CBP. Lactate suppresses SIRT1’s deacetylase activity via the Hippo/YAP pathway, leading to HMGB1’s acetylation and relocation to the cytoplasm. There, HMGB1 transforms into a cytokine, responding to inflammatory stimuli. Lactylated/acetylated HMGB1 is secreted from macrophages via exosomes, increasing endothelial permeability and exacerbating inflammation (56, 57). However, currently, there is no direct evidence of SIRTs regulating lactylation levels of histone/non-histone proteins (such as HMGB1) in macrophages. This could potentially serve as a new research direction for exploration. Lee et al. found that the lack of SIRT2, by inhibiting the activation of NF-κB in macrophages stimulated by lipopolysaccharide (LPS), increases the expression and activity of iNOS, NO, and the generation of reactive oxygen species (ROS), indicating a phenotype of M1 polarization (58). In bone marrow-derived macrophages (BMDMs) stimulated by LPS, pharmacological intervention to enhance intracellular SIRT2 activity led to a decreased acetylation of α-tubulin, subsequently reducing the formation of the NLRP3 inflammasome and presenting an anti-inflammatory phenotype (59). Heinonen and colleagues observed that mice with dual deficiency in SIRT2/3 exhibited enhanced immune killing capacity, protecting the mice from endotoxin harm through increased macrophage cytotoxicity and phagocytosis, alongside increased cytokine secretion. Moreover, BMDMs isolated from mice with a dual deficiency in SIRT3/5, upon stimulation with LPS, exhibited an enhanced inflammatory signaling pathway and increased production of inflammatory cytokines, displaying a pro-inflammatory phenotype (60, 61).

Dendritic cells play a pivotal role in innate immunity, functioning as crucial agents of antigen presentation and immunoregulation. These cells present captured exogenous antigens to T cells and secrete costimulatory molecules and cytokines, fostering the activation and differentiation of T cells (62). SIRTs act as a regulator, bridging innate immune responses with adaptive immunity. For example, within dendritic cells, SIRT1 modulates the release of cytokines IL-12 and TGF-β1 through a HIF1α-dependent pathway, orchestrating the differentiation of CD4+ T cells. In mice injected with SIRT1-deficient dendritic cells, the generation of Treg cells, which suppress excessive immune responses, was diminished, while the development of Th1 cells, which drive cell-mediated immune responses, was spurred, culminating in an enhanced T cell-mediated anti-inflammatory reaction (63). Three years later, Martin et al. arrived at a similar conclusion: treatment of septic mice with the SIRT1-specific inhibitor EX-527, by altering the expression ratio of pro-inflammatory/anti-inflammatory costimulatory molecules (CD80, CD86) and cytokines in dendritic cells, ultimately reduced the Treg cell/activated T cell ratio and reversed the compromised immune state as well as the tolerance of CD4+ and CD8+ T cells to specific antigens (64). While the majority of researchers view SIRT1’s impact on T cell immune function as anti-inflammatory, a position substantiated in various non-septic diseases (65, 66), Zhao et al. discovered contrary results in a septic immunosuppression mouse model. By enhancing the expression of NAD+/SIRT1 in T cells during septic immunosuppression, they reversed the exhaustion of CD4+ and CD8+ T cells, tilting the immune balance back towards a pro-inflammatory response, with increased pro-inflammatory cytokines in the blood and a reduced bacterial load (67). These findings suggest that SIRT1’s effect on T cell function during sepsis may vary, and even be opposing, depending on the phase of the disease, indicating that the deeper mechanisms at play warrant further exploration.

Neutrophils in sepsis, besides phagocytic activity and release of anti-inflammatory substances, also release neutrophil extracellular traps (NETs) which capture bacteria. Numerous studies have shown promising roles of HDACs in regulating NET formation (NETosis) (68). However, the specific regulatory effects of SIRTs, important members of the HDAC family, on neutrophils and NETosis in sepsis have been minimally explored, warranting further attention and confirmation. Moreover, the regulatory capabilities of SIRTs on B cells and NK cells in sepsis remain uncharted territory, which also deserves investigation by researchers.

Endothelial cells (ECs) constitute the vital component of the vascular endothelium, playing a pivotal role in preserving vascular function and barrier integrity. Moreover, as non-immune cells, they possess substantial immunological functions that should not be overlooked. In sepsis, inflammatory mediators, bacterial toxins, and activated immune cells can directly or indirectly inflict damage upon endothelial cells, thereby compromising endothelial barrier function. This can result in heightened vascular permeability, plasma leakage, and tissue edema. Simultaneously, endothelial cells become engaged in the inflammatory response and upregulate the expression of cell surface adhesion molecules like ICAM-1, VCAM-1, and E-selectin. Additionally, the damaged endothelial cells can activate platelets and the coagulation system, culminating in thrombosis that further exacerbates vascular obstruction and organ damage. The Sirtuin family plays a protective role in endothelial cells through multiple mechanisms, in this section, we will focus on their anti-inflammatory effects. Both SIRT1 and SIRT2 can dampen the inflammatory responses of endothelial cells during sepsis through deacetylation, reducing the secretion of NF-κB-dependent adhesion molecules. This, in turn, ameliorates the adherence and aggregation of leukocytes and platelets on the endothelial surface of microcirculation, improving microcirculatory dysfunction (69, 70). SIRT3 is recognized as a pivotal mediator in protecting endothelial cells from mitochondrial oxidative stress. Investigations in septic rat aortic endothelial cells and LPS-stimulated HUVECs have demonstrated that upregulation of SIRT3 expression can enhance AMP-activated protein kinase (AMPK) phosphorylation, inhibiting the activation of the NF-κB and NLRP3 pathways and the release of pro-inflammatory cytokines. This prevents the inflammatory damage to vascular endothelium and increases the survival rate of mice with sepsis (71). The role of SIRT4 in sepsis has been a subject of debate. Recent studies have identified miR-15b-5p and PCSK9 as key mediators in endothelial cell damage during sepsis. Inhibitors of these molecules can reverse the LPS-induced downregulation of SIRT4 expression, attenuate the inflammatory cascade triggered in endothelial cells by LPS, and mitigate their damage and death. This suggests that the functional effects of miR-15b-5p and PCSK9 can, at least in part, be attributed to their ability to modulate SIRT4 expression (72). Earlier research also found that SIRT4 could mitigate the LPS-induced expression of pro-inflammatory cytokines and adhesion molecules in HUVECs by blocking the nuclear translocation of NF-κB p65 (73). Indeed, in addition to the anti-inflammatory effects emphasised in this section, SIRTs also play a role in maintaining endothelial barrier function, preventing endothelial cell apoptosis, and regulating oxidative stress (74). SIRT1 can decrease endothelial tight junction permeability, possibly via RhoA/ROCK signaling pathway (75). SIRTs can inhibit endothelial cell apoptosis by regulating apoptosis-related protein such as caspase-3 (76), thereby protecting endothelial cells from sepsis-induced damage. SIRTs can alleviate oxidative damage to endothelial cells by activating antioxidant response elements, regulating mitochondrial function, and inhibiting oxidative stress pathways, which will be highlighted later in the article. SIRTs play a significant role in reducing sepsis-related multiple organ dysfunction syndrome (MODS) by targeting endothelial cells (**Table 1**).

**Table 1:** Immune/Non-immune Cells and SIRTs.

Cells		SIRTs	Regulatory mechanisms of SIRT	Reference
Immune cells	MDSC	SIRT1	siSIRT1 reduces immunosuppressive effect by enhance mTOR and HIF-1α pathway	(77, 78)
	Macrophage	SIRT1	Directs NF-kB p65 deacetylation, regulates the shift of the inflammatory phenotype	(54, 55)
			Binds to deacetylated lysine residues (28-30) of HMGB1 to forms a stable intranuclear complex, inhibits the release of HMGB1 and mitigates inflammatory responses.	(57)
		SIRT2	Attenuates the acetylation degree of intracellular α-tubulin and reduces the formation of NLRP3 inflammasomes	(59)
		SIRT3	Protects mitochondria and regulates cellular metabolism to maintain a low inflammatory response	(79, 80)
		SIRT4	Mediates the transformation of energy metabolism modes and ends the endotoxin tolerance phase of sepsis	(81)
		SIRT5	Competitive inhibition of SIRT2 on NF-κB P65 deacetylation and enhanced pro-inflammatory response	(49)
		SIRT6	Cooperates with SIRT1, supports the shift of inflammatory phenotype from M1 to M2 in a metabolic manner	(82)
	DC	SIRT1	Affects the product ratio of pro-/anti-inflammatory substance to program the differentiation of T lymphocyte	(63, 64)
	T lymphocyte	SIRT1	Suppress pro-inflammatory response/reverses effector cell depletion during the immunosuppressive phase of sepsis	(66, 67)
Non-immune cells	Endothelial cell	SIRT1、2	Attenuate cell inflammatory response and reduces the secretion of NF-κB-dependent adhesion molecules by deacetylation, protects glycocalyx	(69, 70, 83)
		SIRT3	Inhibits the activation of NF-κB and NLRP3 pathways and the release of pro-inflammatory cytokines by AMPK phosphorylation	(71)
		SIRT4	Attenuates the expression of pro-inflammatory cytokines and adhesion molecules by blocking the nuclear translocation of NF-κB p65	(72, 73)
		SIRT6	Regulates the expression of genes related to inflammation, vascular remodeling, and angiogenesis	(48)

### 2.2 SIRTs and immune cell energy metabolism

#### 2.2.1 Immuno-metabolic reprogramming in sepsis

In the 1920s, Otto Warburg first elucidated the phenomenon known as the Warburg effect, highlighting that tumor cells primarily utilize aerobic glycolysis to consume large amounts of glucose rapidly for ATP production, facilitating rapid proliferation (84). Similarly, during the inflammatory phase of sepsis, immune cells enhance aerobic glycolysis not only by directly upregulating cytosolic enzymes involved in glycolysis but also through various pathways. This shift allows for rapid energy production and supports immune cell functions such as cell migration, cytokine production, and phagocytic activity. Furthermore, metabolic reprogramming also affects immune cell signaling and inflammation regulation. Metabolites can also influence the polarization state of immune cells, thereby affecting their ability to respond to infections, for example, the accumulation of lactate and the formation of an acidic environment, potentially negatively affecting immune cell function (39, 56). Among these, pathways closely associated with SIRTs include the activation of the mTOR/HIF-1α/GLUT1 pathway to promote anaerobic glycolysis metabolism (85, 86) and the inhibition of AMPK and its mediated metabolic pathways, which reduce mitochondrial biogenesis and fatty acid β-oxidation (87). During the anti-inflammatory response phase, an increased influx of fatty acids into mitochondria is observed, with the PGC-1 family orchestrating an enhanced mitochondrial fatty acid oxidation (88). The activation of pathways associated with PGC-1 can alleviate tissue and organ damage caused by the inflammatory response in sepsis (89). SIRTs play a crucial role at numerous junctures in immune metabolism and the transition between inflammatory phenotypes.

#### 2.2.2 The key role of metabolic regulatory network in sepsis

**Figure 1:**
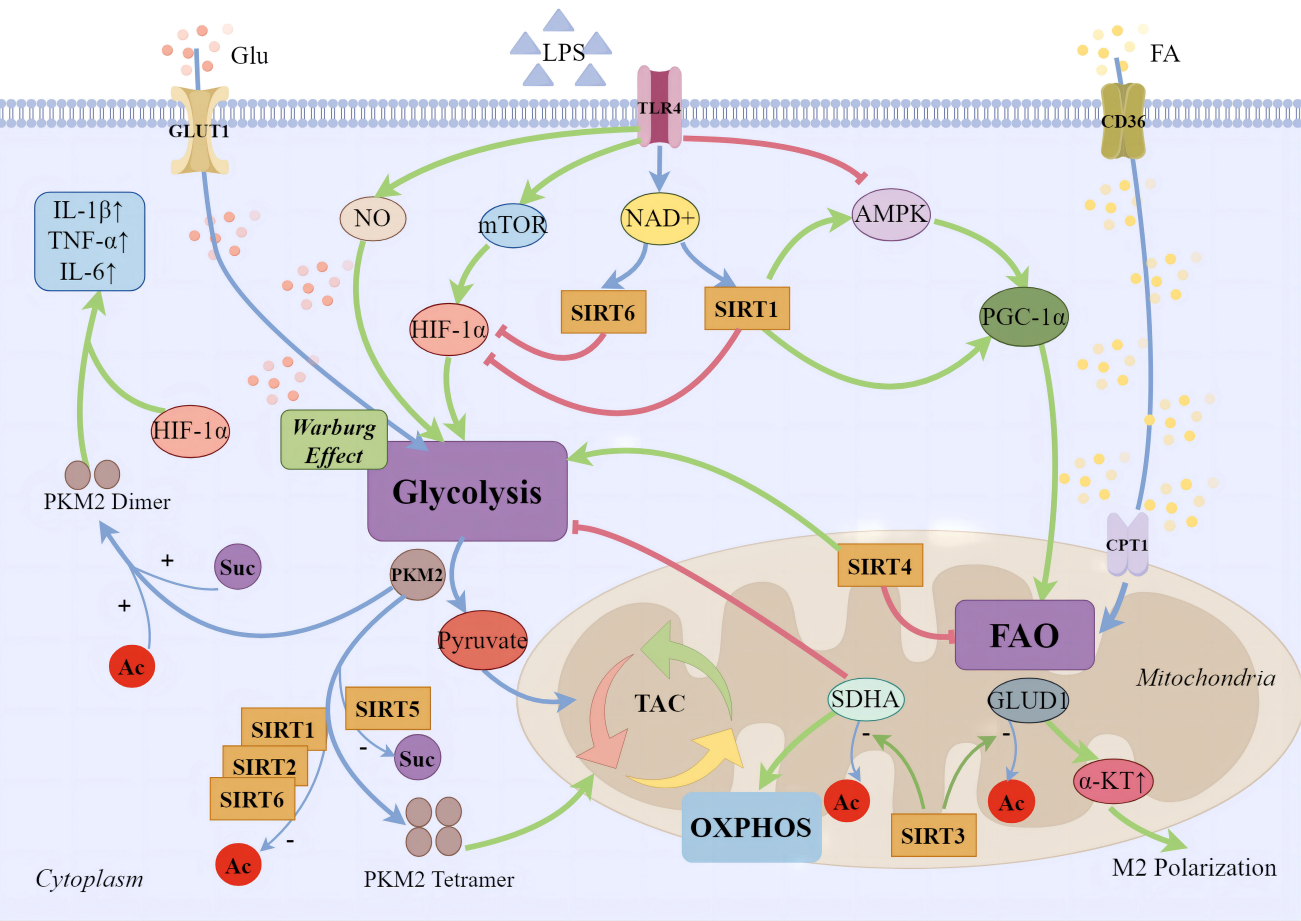
SIRTs and cellular energy metabolism. During the early phases of sepsis-induced inflammatory response, immune cell migration, phagocytosis, bactericidal action, and regeneration processes demand a significant energy expenditure. Apart from the direct upregulation of key aerobic glycolysis enzymes in the cytoplasm, several major pathways have been identified: 1) Pro-inflammatory phase macrophages produce large amounts of NO, inhibiting mitochondrial oxidative respiration chain and increasing glycolytic metabolism; 2) Glycolytic metabolism is promoted through the activation of the mTOR/HIF-1α/GLUT1 pathway; 3) Inhibition of AMPK and its mediated metabolic pathways reduces mitochondrial biogenesis and fatty acid β-oxidation. During the anti-inflammatory phase, increased expression of fatty acid transporter CD36 on the cell membrane and CPT1 on the mitochondrial surface leads to enhanced mitochondrial fatty acid oxidation, mediated by the PGC-1 family. SIRT1 supports anti-inflammatory fatty acid oxidation in immune cells by regulating PGC-1α, while SIRT6 inhibits aerobic glycolysis mediated by silencing HIF-1α target genes. SIRT3 promotes the deacetylation of GLUD1 in mitochondria, facilitating the production of α-ketoglutarate, supporting macrophage M2 polarization. The deacetylation of complex II’s SDHA improves the efficiency of the oxidative phosphorylation chain, thereby reducing the production of ROS. SIRT1, SIRT2, and SIRT6 enhance the tetramerization of PKM2 through deacetylation, reducing the Warburg effect. Moreover, SIRT5 regulates PKM2 by desuccinylation. Dimerized PKM2 enters the nucleus, cooperates with Hif-1α to bind to the promoter region of IL-1β, significantly enhancing the transcription of the pro-inflammatory factor IL-1β and augmenting the glycolytic function of macrophages.

In the intricate immune-metabolic landscape of sepsis, the AMPK/SIRT1/PGC-1α axis orchestrates a pivotal regulatory network that modulates cellular inflammatory responses. Serving as a metabolic sensor, AMPK enhances the activity of SIRT1 by elevating cellular NAD+ levels. In turn, SIRT1 supports the anti-inflammatory lipid oxidation in immune cells by regulating PGC-1α (90). In LPS-stimulated THP-1 macrophages, the transition from an early pro-inflammatory to a late anti-inflammatory phase hinges on Nampt-dependent NAD+ synthesis. During the later phase, both SIRT1 and SIRT6 expressions are elevated. SIRT1 supports fatty acid oxidation pathways by directly binding to and activating PGC-1, while SIRT6 suppresses aerobic glycolysis mediated by HIF-1α through the silencing of its target genes, thus jointly coordinating the metabolic shift from glucose to fatty acid utilization (82). During the anti-inflammatory phase of sepsis, fatty acid oxidation becomes the predominant fuel source, replacing glycolysis. SIRT1 and RelB sequentially activate the transcription of SIRT3, enhancing fatty acid oxidation while concurrently inhibiting oxidative phosphorylation and mitochondrial biogenesis. This shift renders immune cells more reliant on fatty acid oxidation (91). Researchers have revealed SIRT3 participates in facilitates the deacetylation of mitochondrial glutamate dehydrogenase 1 (GLUD1), increasing its activity in glutamate breakdown, promoting the production of alpha-ketoglutarate, and through the control of glutamine breakdown leading to alpha-ketoglutarate accumulation, supports the M2 polarization of macrophages (79). SIRT3 can deacetylate and activate several key enzymes within mitochondria, such as SDHA (succinate dehydrogenase), a component of complex II. This regulation increases the efficiency of the oxidative phosphorylation chain, reduces electron leakage, and thereby lowers ROS production. In LPS-stimulated macrophages, overexpression of SIRT3 through SDHA deacetylation, mitigates sepsis-associated aerobic glycolysis (80).The role of SIRT4 in inflammatory immune responses is not entirely clear yet. However, studies have found that during the endotoxin tolerance phase in THP-1 cells, SIRT4 reverses the anti-inflammatory phase’s fatty acid oxidation back to the pro-inflammatory phase’s glucose oxidation and increases the expression of fatty acid synthase (81). Therefore, for macrophages, SIRT4 may be a counteracting factor against their anti-inflammatory response, concluding the endotoxin tolerance phase of sepsis. PKM2 is a key enzyme in glycolysis, with its deacetylated tetrameric form supporting the TCA cycle. In LPS-stimulated macrophages, PKM2 mainly exists in highly acetylated dimeric/monomeric forms, promoting the Warburg effect (92). SIRT1 upregulation reduces PKM2 acetylation, preventing its nuclear accumulation and alleviating inflammatory responses in sepsis-induced alveolar epithelial cells (93). SIRT2 and SIRT6 also deacetylate PKM2, promoting its tetrameric functionality (94, 95). SIRT5 plays a crucial role in regulating PKM2 by preventing its succinylation-induced transition to a dimeric form. This regulation leads to a downregulation of IL-1β transcription by reducing the cooperation between PKM2 and Hif-1α, which binds to the promoter region of IL-1β. Additionally, it enhances the aerobic glycolysis function of macrophages (96). Additionally, Wang et al. showed that lactate increases PKM2 lactylation, inhibiting its tetramer-to-dimer transition. This inhibition of the Warburg effect promotes the transition of pro-inflammatory macrophages to a reparative phenotype. However, the involvement of SIRTs in this process is currently unknown and requires further exploration (97) ( **Figure 1**).

### 2.3 SIRTs and oxidative stress damage

During the onset of sepsis, an inflammatory response is triggered, activating immune cells that release a vast array of pro-inflammatory cytokines, chemokines, and cytotoxins. This inflammatory milieu affects the mitochondrial electron transport chain (ETC), leading to the generation of significant levels of ROS and reactive nitrogen species (RNS) (98). Immune cells, through phagocytosis and respiratory bursts, produce substantial amounts of ROS (99). This, in turn, can have potential benefits in the killing and clearance of pathogens by modulating immune cell signaling and inflammation regulation. ROS have the ability to directly eliminate pathogens, such as bacteria and fungi, by oxidizing their cell membranes and DNA. Furthermore, ROS can regulate the production of cytokines and inflammation by immune cells, thereby influencing the intensity and duration of the inflammatory response. Moderate oxidative stress can enhance the ability of immune cells to kill and clear pathogens, but excessive oxidative stress may cause harm to immune cells and lead to inflammation-induced tissue damage (100).

Under normal physiological conditions, cells employ enzymatic antioxidants such as superoxide dismutase (SOD), catalase (CAT), and glutathione peroxidase (GPx), as well as non-enzymatic antioxidants like vitamins C and E, and reduced glutathione (GSH), to neutralize excess ROS (101). However, when the production of ROS surpasses the capacity of these endogenous antioxidant mechanisms to clear them, oxidative stress ensues. The high levels of ROS damage cellular structures and impair the function of vital organs, contributing to organ dysfunction. ROS also activate pro-inflammatory signaling pathways, leading to the release of more pro-inflammatory substances and amplifying the inflammatory response. Additionally, ROS disrupt endothelial cell function, causing increased vascular permeability (102). This allows inflammatory cells and fluid to enter tissues, leading to edema and organ dysfunction (103, 104). Oxidative stress also affects immune cell function and responsiveness, impairing their ability to clear pathogens. Overall, targeting oxidative stress and restoring redox balance may have therapeutic potential in mitigating the harmful effects of sepsis (105). SIRTs play a pivotal role in mediating cellular responses to oxidative stress. By modulating cellular metabolism, enhancing antioxidant defense mechanisms, and maintaining mitochondrial function, SIRTs contribute significantly to the regulation of ROS levels and prevention of oxidative stress damage during sepsis.

SIRTs such as SIRT1, SIRT2, and SIRT3 possess the capability to deacetylate the FOXO3a transcription factor, culminating in its activation (106, 107). The activation of FOXO3a triggers the expression of antioxidative genes, including SOD and various antioxidative enzymes, thereby providing a robust defense against ROS-induced cellular damage. Recent findings suggest that pharmacological enhancement of SIRT1 expression within cells can significantly attenuate the acetylation levels of FOXO3a induced by cisplatin in HK-2 cells, simultaneously elevating the expression of the antioxidative enzyme GPx. This leads to a reduction in ROS generation and the inhibition of lipid peroxidation and cell death (108). Moreover, SIRT1 is known to modulate cellular antioxidative defenses through the NRF2 pathway. NRF2, a transcription factor, remains inactive in the cytoplasm bound to Keap1 under resting conditions. Upon oxidative or chemical stress, NRF2 dissociates from Keap1, translocates to the nucleus, and activates the expression of antioxidative and cell-protective genes. SIRT1 facilitates the release of NRF2 by deacetylating Keap1, thereby initiating the expression of downstream antioxidative genes (109). Zhao and colleagues have demonstrated that pharmacological interventions targeting the SIRT1/AKT/NRF2 pathway in LPS-induced C2C12 myotubes upregulate the expression of antioxidative genes and proteins, including NRF2, SOD2, CAT, and GPX4, while exhibiting an inverse expression pattern for Keap1 protein. This modulation ameliorates oxidative stress-induced damage and apoptosis (110). Additionally, SIRT1 enhances the NOX4-NRF2 axis by directly or indirectly deacetylating NRF2, promoting the expression of downstream antioxidative enzymes. This strengthens endothelial cell resistance against oxidative stress-induced pathological activation and mitochondrial pathway apoptosis (111). SIRT2 and SIRT6 also exhibit antioxidative functions via the NRF2 pathway (112, 113). The activation of the SIRT1/PGC-1α axis further signifies the awakening of antioxidative defense mechanisms, mitigating mitochondrial oxidative stress. Liu and collaborators have observed that in cerebral tissue of septic mice treated with cecal ligation and puncture (CLP), the activation of the SIRT1/PGC-1α pathway not only reversed mitochondrial structural damage but also mitigated the elevation of ROS and malondialdehyde (MDA) levels, and the restoration of SOD levels has been achieved. This also significantly improved the activity of mitochondrial respiratory chain complexes, markedly alleviating sepsis-associated encephalopathy (114).

SIRT3 is highly active within mitochondria and modulates mitochondrial quality control homeostasis at multiple junctures. A salubrious mitochondrial architecture contributes to the maintenance of oxidative phosphorylation efficiency and the reduction of ROS production. Beyond its SIRT1-analogous functions, such as promoting mitophagy and exerting antioxidative stress effects through pathways like SIRT3/FOXO3a and AMPK/PGC-1α/SIRT3 (115, 116), SIRT3 also directly deacetylates and activates enzymes associated with antioxidation, enhancing the scavenging capacity for ROS. The application of melatonin upregulates the expression of SIRT3, triggering the deacetylation of SOD2, augmenting its activity, and diminishing oxidative stress injury in the small intestinal tissues of septic model rats and in septic pulmonary epithelial cells—thereby protecting mitochondrial function (117). SIRT3 further enhances the efficiency of the oxidative phosphorylation chain by deacetylating the pivotal enzyme SDHA, mitigating electron leak and consequently reducing the generation of ROS during sepsis and ischemia-reperfusion (80). Researchers have substantiated that increased expression and activity of SIRT3 result in the deacetylation and enzymatic activation of mitochondrial fatty acid oxidation enzyme (LCAD), which, by modulating fatty acid oxidation, aids SIRT3 in the maintenance of energy metabolic equilibrium and the diminution of ROS production (118). Tubeimoside I (TBM) treatment reverses the significant decrease in vascular endothelial SIRT3 expression induced by sepsis, reduces oxidative stress by decreasing NOX2 and Ac-SOD2/SOD2 levels and alleviates cell apoptosis by inhibiting cleaved caspase3 and Bax/Bcl-2 (119). Similar results were obtained in a study by Wu, where it was found that Polydatin (PD) can also reverse the decreased expression of SIRT3 in endothelial cells induced by LPS. PD enhances SIRT3 deacetylation levels, reduces ROS production, and inhibits mitochondrial dysfunction and subsequent endothelial barrier dysfunction (74).

Beyond its deacetylation capabilities, SIRT5 distinguishes itself within the sirtuin family through its unique desuccinylation and deglutarylation activities, playing a crucial role in metabolic regulation and antioxidant stress response. SIRT5, by mediating lysine desuccinylation, stabilizes mitochondrial metabolism following subarachnoid hemorrhage in mice, thereby reducing the production of ROS (120). Additionally, SIRT5 desuccinylates and activates SOD1, enhancing its ROS scavenging capacity (121). Researchers have also discovered that SIRT5 activates isocitrate dehydrogenase 2 (IDH2) and glucose-6-phosphate dehydrogenase (G6PD) through desuccinylation and deglutarylation, respectively. This activation leads to the production of NADPH, supporting the maintenance of GSH for ROS clearance and safeguarding cells from oxidative damage (122). In TNF-α-induced umbilical cord blood-derived mesenchymal stem cells (UCB-MSCs), silencing SIRT5 resulted in decreased fatty acid β-oxidation and accumulation of ROS within mitochondria, consequently exacerbating the senescence of UCB-MSCs (123). Thus, SIRT5 plays a pivotal role in maintaining cellular redox balance and mitigating oxidative stress-induced cellular damage and aging.

SIRT6 employs a multifaceted approach to combat oxidative stress, engaging not only in the modulation of metabolic pathways and anti-inflammatory responses but also in the regulation of DNA repair and the expression of antioxidative genes. These actions collectively aid in maintaining cellular redox balance and safeguarding cells from oxidative damage. SIRT6 protects retinal ganglion cells from hydrogen peroxide-induced apoptosis and oxidative stress through the NRF2/ARE signaling pathway (113). Oxidative stress can lead to DNA damage, and SIRT6 plays a critical role in promoting DNA repair. Cells deficient in SIRT6 exhibit heightened sensitivity to oxidative stress and diminished DNA repair capabilities, whereas SIRT6 knockout mice display symptoms of premature aging (124). Overexpression of SIRT6, by inhibiting the JAK2/STAT3 pathway and enhancing SOD activity, boosts the capacity to clear ROS, thereby suppressing oxidative stress and the growth of glioblastoma cells (125).

**Figure 2:**
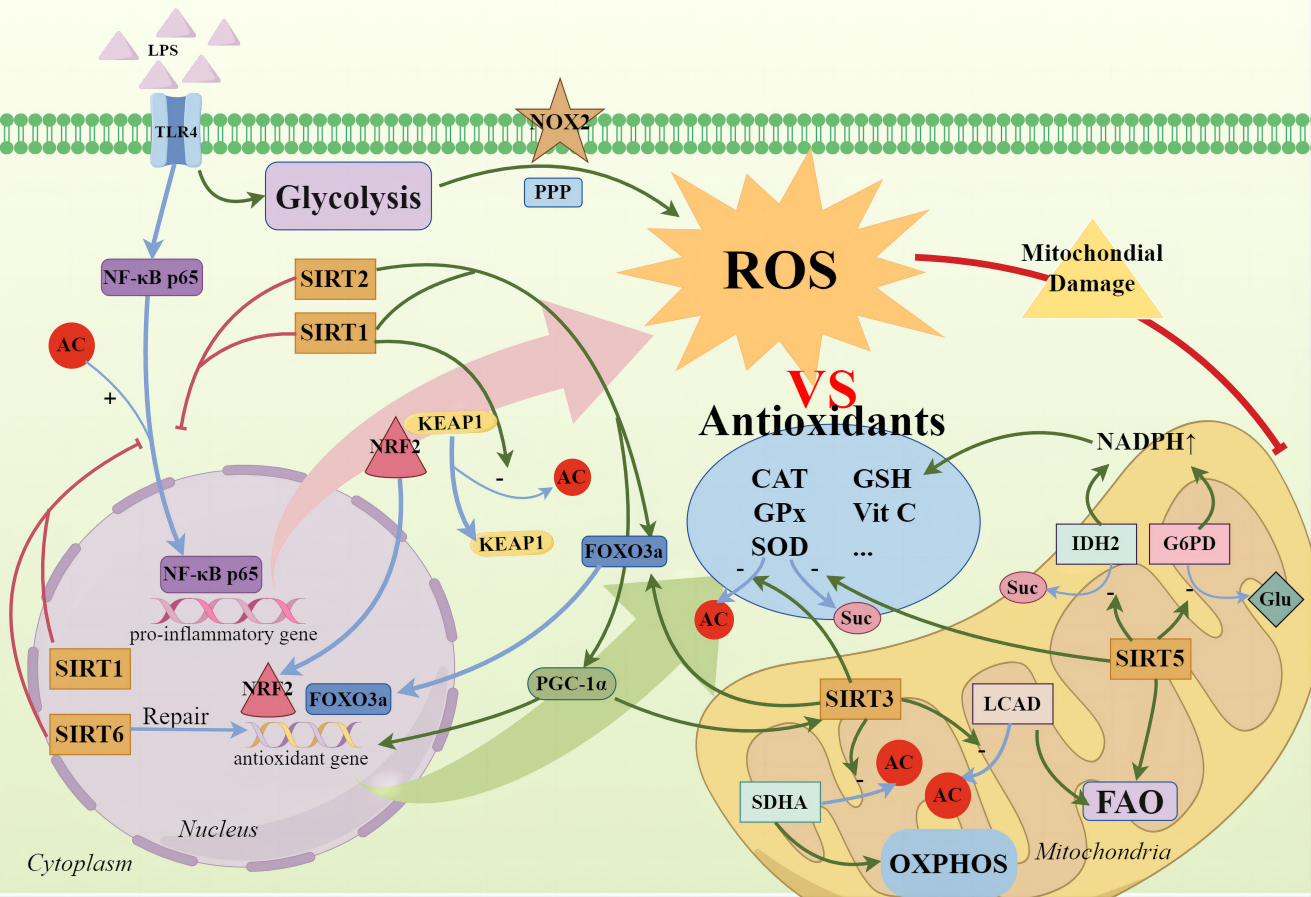
SIRTs and cellular oxidative stress. During the high-inflammatory phase, glycolysis not only rapidly supplies ATP but also significantly intensifies the PPP, activating NOX2 to produce ROS. Concurrently, the nuclear translocation of NF-κB p65 promotes the expression of pro-inflammatory genes, exacerbating ROS production. Cells employ antioxidative enzymes—such as SOD, CAT, GPx—and non-enzymatic antioxidants, including vitamins C and E, and GSH, to neutralize excess ROS. SIRTs not only directly deacetylate to enhance SOD activity but also promote the activation of the FOXO3a transcription factor, with SIRT1, SIRT2, and SIRT3 being notable contributors. SIRT1 mediates the deacetylation of Keap1 to release NRF2, allowing its translocation to the nucleus to initiate the expression of downstream antioxidant genes. The activation of the SIRT1/PGC-1α axis also signifies the activation of antioxidant defense mechanisms, mitigating mitochondrial oxidative stress. Additionally, SIRT3 enhances the deacetylation of SDHA and LCAD, regulating oxidative phosphorylation and fatty acid oxidation to reduce ROS production. SIRT5 activates IDH2 and G6PD through desuccinylation and deglutarylation respectively, generating NADPH to help maintain the reductive state of GSH for ROS clearance and cellular protection against oxidative damage. Furthermore, SIRT6 promotes the repair of antioxidative DNA damage.

SIRTs play a significant role in regulating ROS and promoting antioxidation; however, their protective capabilities may be constrained in the face of severe oxidative stress and inflammation induced by sepsis. Therefore, it is necessary to combine other treatments to effectively manage the oxidative stress associated with sepsis (**Figure 2**).

## 3 SIRTs and sepsis-induced MODS

As previously described, SIRTs act as critical regulatory factors for both immune and non-immune cells’ inflammatory stress responses, mediating the pathophysiological process of sepsis by coordinating metabolism and immunity, among other aspects. In infectious diseases, there is a notable aberration in the expression of SIRTs. For instance, the extent of reduction in serum levels of SIRT3 can differentiate between sepsis and septic shock patients and is well correlated negatively with the SOFA score. Moreover, SIRT3 holds promise as a marker for diagnosing and prognosticating sepsis, given its ability to predict mortality in septic patients (126). The Sirtuin family primarily functions through deacetylation, with its activity being regulated by factors such as the NAD+/NADH ratio, SIRT binding proteins, and post-translational modifications (127). Furthermore, SIRTs are extensively involved in epigenetic modifications of multiple organs under sepsis conditions, impacting the respective organs. In this section, we will summarize the significant role of SIRTs in sepsis-induced damage across different organ systems, aiming to highlight potential targets for future research on the treatment of sepsis-induced MODS (**Table 2**).

**Table 2:** SIRTs involved in the process of multiple organ damage in sepsis.

Organ	SIRTs	Effects	Reference
Lung	SIRT1	Mediates the activation of inflammasome. Affects the integrity of tight and adherent connections	(128–130)
	SIRT3	ADSC-derived exosomes promote autophagic activation through SIRT3/AMPK signaling	(131)
Liver	SIRT1	MCPIP1 promotes SIRT1 expression to inhibit p65 acetylation, alleviates septic liver injury	(132)
		Impairs NF-κB activity, results to increased susceptibility to endotoxin damage	(133)
Kidney	SIRT1	Attenuation of pyroptosis in sepsis kidney injury through the SIRT1/PGC-1α/Nrf2 signaling pathway	(134)
		Deacetylates HMGB1 and inhibits downstream inflammatory signaling pathways	(135, 136)
		Specifically induces deacetylation of the autophagy-Beclin1, mediates autophagy in sepsis kidney injury	(137)
	SIRT3	alleviates oxidative stress and downregulates the expression of inflammatory mediators	(138)
		Promotes mitochondrial fusion and attenuates mitochondrial damage and apoptosis	(139)
	SIRT5	Enhances the phosphorylation of AMPK and alleviates mitochondrial dysfunction	(140)
	SIRT6	Expression levels synchronously fluctuate with LC3B protein over time, enhancing autophagy and diminishing apoptosis	(141)
Heart	SIRT1	Augments the antioxidative capacity of FOXO1 and resistance to endoplasmic reticulum stress, enhances the deacetylation status of NF-κB.	(142)
	SIRT3	Activates the AMPK pathway, improves mitochondrial biogenesis, mediates p53 deacetylation to alleviate oxidative stress damage and ferroptosis	(143, 144)
Intestine	SIRT1	Participates in the regulation of apoptosis and protect cell viability	(145, 146)

### 3.1 SIRTs and sepsis-induced lung injury

In the context of sepsis-induced lung injury, SIRT deficiency can lead to severe inflammatory pulmonary damage. Gao et al. reported that mice with SIRT1 deficiency after CLP showed increased inflammasome activity, resulting in elevated production of pro-inflammatory mediators such as ICAM-1 and high-mobility group box 1 (HMGB1). This was accompanied by further disruption of tight junctions and adherens junctions, as evidenced by a marked decrease in the expression of lung Claudin-1 and vascular endothelial cadherin (128). The long non-coding RNA OIP5-AS1 exhibits a trend of expression that parallels SIRT1 during sepsis-induced lung injury. The regulatory axis comprising lncRNA OIP5-AS1, SIRT1, and miR-128-3p is implicated in the pathogenesis of septic lung injury. Overexpression of lncRNA OIP5-AS1 is associated with a concurrent increase in SIRT1, leading to decreased expression of inflammatory factors and amelioration of lung edema, epithelial cell apoptosis, infiltration of myeloperoxidase (MPO)-marked polymorphonuclear neutrophils (PMNs), and the inflammatory response induced by CLP *in vivo* (129). miR-199a can target the regulation of SIRT1 in alveolar macrophages, and its downregulation may protect against sepsis-induced lung injury by upregulating SIRT1 through inhibition of excessive inflammatory responses and suppression of apoptosis in lung tissue (130). Adipose-derived stem cell (ADSC) secreted exosomes also offer protection against sepsis-induced lung injury. Shen et al. discovered that exosomes derived from ADSCs are enriched with circular RNA Fryl (circ-Flyl), which promotes autophagy activation via the SIRT3/AMPK signaling pathway. Activation of autophagy can mitigate sepsis-induced lung injury by reducing cell apoptosis and the expression of inflammatory factors (131).

### 3.2 SIRTs and sepsis-induced liver injury

The reduction of NAD+ impairs the function of SIRTs in sepsis and leads to mitochondrial dysfunction (147). Studies by Cao et al. also support this: increasing NAD+ levels can protect against sepsis-induced multiorgan damage by preventing mitochondrial dysfunction and oxidative stress through SIRT3 signaling (76). The impact of NAD+ levels on the regulatory role of SIRTs in different organs is worth exploring. Glutamine can upregulate the protein synthesis of SIRT4 and activate SIRT4 by increasing the level of NAD+, thereby reducing the acetylation of heat shock protein (HSP) 60 and promoting the assembly of the HSP60-HSP10 complex. This complex maintains the activity of electron transport chain complexes II and III, sustaining ATP production and reducing the release of reactive oxygen species. The restoration of mitochondrial complex activity and maintenance of cellular energy metabolism through the regulation of HSP60-HSP10 complex assembly can play a protective role in the liver during sepsis (148). The liver is the organ with the highest expression of NAD+ enzymes, and as NAD+-dependent enzymes, the role of SIRTs in endotoxin-induced liver damage remains unclear to date. On one hand, researchers have discovered that the Monocyte Chemotactic Protein-1 Induced Protein 1 (MCPIP1), acting as a negative regulatory factor in inflammation, can control p65 acetylation by promoting the expression of SIRT1. Additionally, it regulates the transcription of SIRT1 by modulating the interaction between microRNA-9 and the 3’-untranslated region of SIRT1 mRNA, thereby mitigating the inflammatory response and sepsis-induced liver damage (132). Interestingly, a study by Cui et al. suggested that SIRT1 might also have a detrimental effect on sepsis-induced liver damage. Under LPS/TNF-α stimulation, the deacetylation of NF-κB p65 by SIRT1 impaired NF-κB activity in liver cells, increasing susceptibility to endotoxin damage. This phenomenon could be attributed to SIRT1’s involvement in the extensive energy metabolism in the liver and its dependence on NAD+ (133). Many studies have investigated the therapeutic potential of natural or synthetic compounds targeting SIRTs pathway for the treatment of sepsis-induced liver injury. We will provide detailed explanations in the subsequent section on pharmaceutical treatments.

### 3.3 SIRTs and sepsis-induced kidney injury

Amidst sepsis-induced renal injury, there is a pronounced depletion of NAD+ levels, which inversely correlates with the extent of kidney dysfunction (149). NAD+ serves as the critical substrate for maintaining the deacetylase activity of SIRT1. As an intermediary of NAD+, β-Nicotinamide Mononucleotide (NMN) facilitates the restoration of NAD+ levels, significantly ameliorating oxidative stress and cellular apoptosis induced by LPS, thereby mitigating kidney injury. He and colleagues reveal that inhibitors of SIRT1 diminish the protective effects of NAD+, decreasing nuclear accumulation of NRF2, consequently exacerbating septic kidney injury (150). Research by Ling and others highlights that in septic damage, the inhibition of miR-579-3p by LncRNA GAS5 activates the SIRT1/PGC-1α/NRF2 signaling pathway, reducing cellular pyroptosis in septic kidney injury (134). SIRT1’s ability to regulate transcriptional activity of multiple targets enables it to moderate various metabolic and stress pathways. Given that HMGB1 is a key inflammatory mediator in sepsis, Wei and colleagues have ascertained that SIRT1 can interact with HMGB1 at lysine residues K28, K29, and K30 to effect deacetylation, inhibiting downstream inflammatory signaling and participating in the protection against septic kidney injury (135). Furthermore, Zeng and associates discovered that lncRNA GAS5 forms a regulatory axis with miR-155-5p/SIRT1/HMGB1 in sepsis, with lncRNA GAS5 upregulating SIRT1 to suppress HMGB1 acetylation and release (136). SIRT1 specifically induces deacetylation of the autophagy-related gene Beclin1 at lysine residues K430 and K437 to mediate cell autophagy during septic kidney damage, thereby conferring protection (137). In septic kidney injury models, SIRT3 expression is downregulated; the absence of SIRT3 augments expression of the NLRP3 inflammasome and apoptosis-associated speck-like protein, leading to enhanced oxidative stress, pro-inflammatory cytokine production, and increased apoptosis. SIRT3 itself can attenuate oxidative stress, downregulate IL-1β and IL-18, and protect against renal mitochondrial damage (138), whereas *in vitro* overexpression of SIRT3 in HK-2 cells mitigates injury, a mechanism explained by SIRT3’s promotion of mitochondrial fusion through deacetylation of the upstream regulator i-AAA protease (YME1L1) to alleviate LPS-induced damage and apoptosis in renal tubular epithelial cells (139). In models of septic kidney injury, upregulation of SIRT5 expression enhances phosphorylation of AMPK, alleviating mitochondrial dysfunction in renal tubular epithelial cells, thus diminishing septic kidney injury (140). SIRT6 plays a role in regulating autophagy in septic kidney damage. Microtubule-associated proteins 1A/1B light chain 3B (LC3B) is a protein involved in autophagy. Zhang and colleagues have examined renal SIRT6 and LC3B protein in a mouse LPS model. Their findings suggest a biphasic progression of kidney injury in the LPS model. Within 12 hours post-model induction, levels of SIRT6 and the ratio of LC3B-II to LC3B-I significantly increase, while at 48 hours, they markedly decrease. Additionally, overexpression of SIRT6 downregulates the secretion of TNF-α and IL-6, inhibits apoptosis, and promotes autophagy. Conversely, silencing of the SIRT6 gene yields opposite results (141).

### 3.4 SIRTs and sepsis-induced cardiac injury

Liver X receptors (LXRs), members of the nuclear receptor superfamily, have been revealed to play a role in the pathophysiology of heart failure and are also of significant importance in sepsis (151, 152). Han and colleagues discovered that LXR agonists could suppress myocardial inflammatory cytokines, oxidative stress, endoplasmic reticulum stress protein levels, and myocardial cell apoptosis following CLP in mice. However, these effects were not observed in CLP mice with a SIRT1 deficiency. Mechanistically, these phenomena can be attributed to the LXR agonists enhancing SIRT1 signaling, thereby boosting the antioxidant level of FOXO1 and resistance to endoplasmic reticulum stress, as well as deacetylating NF-κB, which in turn mitigates sepsis-induced myocardial injury and dysfunction (142). SIRT3 and AMPK stimulate mitochondrial biogenesis and increase mitochondrial turnover. In a murine model of LPS-induced cardiac injury, levels of SIRT3 and AMPK decreased, followed by mitochondrial dysfunction and myocardial cell death. Overexpression of SIRT3 activated the AMPK pathway and improved mitochondrial biogenesis (143). Moreover, ferroptosis, a form of cell death associated with oxidative stress and inflammation, has been identified as playing a role in sepsis-related cardiac dysfunction. The ANXA1 small peptide (ANXA1sp) mitigates the overproduction of pro-inflammatory cytokines in myocardial cells, reverses the increase in ROS and MDA, and ameliorates the reduced activity of SOD and GSH, concurrently inhibits iron accumulation while boosting the expression of GPX4, which is considered a key regulator and suppressor of ferroptosis, with its activity serving as a defense against this iron-dependent form of cell death. These effects are mechanistically credited to SIRT3-dependent deacetylation of p53; without SIRT3, these protective phenomena do not occur (144).

## 4 Drugs targeting the SIRTs in sepsis-induced MODS

Research into the SIRTs as targets for sepsis-induced MODS is still at an early stage, yet the field holds promising prospects. As previously indicated, SIRTs may be decisive factors in the outcomes of septic shock and its associated metabolic, immune, and biological reprogramming. In current research, the targeted application of SIRTs is related to the context of the inflammatory response, which undergoes a transition from an early/high inflammatory state to a late/low inflammatory state until it subsides. Meanwhile, SIRTs also undergo corresponding pathophysiological changes. A number of natural or synthetic substances have been identified that can alleviate the extent of sepsis-induced MODS through pathways involving SIRTs. Compared to most libraries used for high-throughput screening, these compounds serve as starting points for the development of isoform-selective and highly efficacious drug-like compounds, demonstrating potential applications in human health and clinical trials, based on their ability to save time and reduce economic costs.

### 4.1 Melatonin

Melatonin, a potent immunomodulatory molecule, has garnered significant interest due to its ability to inhibit the HMGB1 signaling pathway and the activation of TLR4, consequentially preventing the activation of inflammasomes such as NLRP3 and NF-κB. This leads to an upregulation of NRF2 expression. In clinical trials, Taher investigated melatonin as a drug for the treatment of multiple organ damage in sepsis and found that it significantly improved the SOFA scores in the treatment group compared to the placebo group (153). However, clinical trials in this field lack multicenter, large-scale data to support its therapeutic effects in real-world patient populations. Interestingly, melatonin’s functionalities echo the actions of SIRT1, a correlation supported by numerous studies demonstrating melatonin’s capability to alleviate inflammatory responses through SIRT1-mediated effects. Research indicates that melatonin treatment, activating the SIRT1/NRF2 signaling pathway, can mitigate oxidative stress damage, acute neuroinflammation, and apoptosis in the hippocampal DG region of PND7 rats induced by LPS (154). In an endeavor to simulate a septic environment *in vivo*, Zhao et al. utilized a CLP model in male C57BL/6J mice administrated intraperitoneally with melatonin. The results showcased a melatonin-induced decrease in the production of TNF-α, IL-1β, and HMGB1, alongside an elevation in the activities of SOD and CAT, with a concurrent reduction in MDA production. The process was characterized by an upregulated expression of SIRT1 and the B cell lymphoma 2 apoptosis regulator (Bcl-2), and a downregulated expression of FoxO1, p53, NF-κB, and Bax. However, the protective effects of melatonin were nullified upon administering the SIRT1 specific inhibitor EX-527, providing compelling evidence for melatonin’s role in mitigating sepsis-induced brain damage through the activation of SIRT1 signaling (155).

The cardioprotective efficacy of melatonin against sepsis-induced myocardial damage was further substantiated in comparative analyses of control, LPS, LPS + melatonin, and LPS + melatonin + EX-527 groups. These analyses, focusing on myocardial injury markers such as creatine kinase (CK) and creatine kinase-MB (CK-MB) levels, cardiac structure, as well as cellular apoptosis and autophagy scenarios, revealed melatonin’s action through SIRT1 (156). Melatonin treatment promoted Beclin-1 deacetylation and enhanced autophagy in septic hearts, thereby ameliorating cardiac function. Furthermore, melatonin elevated the expression and activity of SIRT1, and the use of EX-527 negated the protective effects of melatonin on Beclin-1 deacetylation and cardiac function. This evidence posits that melatonin could facilitate Beclin-1 deacetylation and improve sepsis-induced autophagy and cardiac function through SIRT1 (157).

Chen et al. delved into the therapeutic implications of melatonin in sepsis-induced liver damage, evaluating markers of glucose metabolism, inflammation, liver functionality, and related signaling pathways. The study unveiled significant amelioration of hepatic SIRT1/STAT3 pathway suppression, hyperglycemia, and hepatic gluconeogenesis by melatonin. Moreover, EX-527 notably detracted from melatonin’s protective stance against sepsis-induced liver damage, hyperglycemia, and STAT3 deactivation, proffering prospects for melatonin’s approach in treating sepsis-induced liver damage through SIRT1-mediated STAT3 pathways (158).

Additionally, the combined usage of melatonin with ascorbic acid demonstrated protective effects against sepsis-induced MODS. The study by Üstündağ et al. showcased that such a combined treatment reduced inflammation and cellular damage markers, improved NO, and vascular endothelial growth factor (VEGF) levels, alongside an increased SIRT1 expression, indicating a synergistic role in alleviating sepsis-induced inflammation, cellular damage, and oxidative stress - an assertion confirmed through histopathological analyses of the heart and kidneys (159).

Recent studies have expanded the spotlight from SIRT1 to include SIRT3 as a crucial participant in the protective effects of melatonin against sepsis-induced MODS. A pivotal study by Ning and colleagues investigated the pathological damage, inflammatory response, oxidative stress, and apoptosis in epithelial cells of the lungs induced by sepsis. The findings reveal that melatonin leverages a SIRT3-dependent pathway to enact deacetylation of SOD2, thereby safeguarding mitochondrial quality control in pulmonary epithelial cells, ultimately mitigating the sepsis-induced damages, inflammation, oxidative stress, and cell apoptosis (117). In parallel, research conducted by Xu et al. underscored the protective role of melatonin in alleviating sepsis-induced intestinal damage. This study demonstrated that melatonin restores the activity and/or expression of SIRT1 and SIRT3 in the small intestine, enhancing the deacetylation process of NF-κB. This process led to a decrease in the levels of inflammatory factors, alongside an increase in the deacetylation and activity of SOD2, which contributes to a reduction in oxidative stress. The use of EX-527 and the SIRT3 selective inhibitor 3-TYP highlighted that the improvements in oxidative stress mediated by melatonin were impeded, elucidating that melatonin’s modulation of oxidative stress inhibition, mitochondrial function protection, and induction of autophagy operates independently from SIRT1 through the upregulation of SIRT3 (160). These pre-clinical stage studies provide a good prospect for future clinical studies by researchers.

### 4.2 Resveratrol

Resveratrol (RSV), a polyphenolic phytoalexin produced by plants in response to stressors like UV radiation, mechanical injury, or fungal infection, has garnered attention for its profound potential in the medical field. Renowned as a potent activator of SIRT1, RSV boasts an array of health-promoting properties, encompassing anti-inflammatory, anti-tumor, and cardiovascular protective effects (161–163). The efficacy of RSV in mitigating microvascular inflammation through the upregulation of endothelial cell SIRT1 levels, consequently diminishing the expression of E-selectin and ICAM-1 as well as alleviating leukocyte and platelet adhesion, has demonstrated protective outcomes in SIRT1-deficient, CLP-induced obese mice models. This effect is notably attenuated following the administration of EX-527 (69).

In the context of septic cardiac injury, RSV also presents promising therapeutic prospects. Research by An et al. focuses on the activation of SIRT1 signaling by RSV, leading to the reduction in the accumulation of neutrophils, TNF-α expression, and myocardial cell apoptosis, thereby conferring protection against septic myocardial injury through the measurement of TNF-α, myeloperoxidase (MPO), SIRT1, acetylated-FoxO1, and Bcl-2 levels in sepsis model mice (164).

In treating septic liver damage, Xu and colleagues propose that RSV preconditioning could lessen liver injury by reducing the levels of serum transaminase and pro-inflammatory chemokines. Mechanistically, *in vitro* experiments demonstrated that SIRT1 inhibits HMGB1 translocation, a phenomenon further substantiated by the increased suppression of HMGB1 translocation with RSV in the absence of SIRT1. *In vivo* analyses reveal a physical interaction between SIRT1 and HMGB1 within the nucleus, with SIRT1 modulating the acetylation of HMGB1 to combat sepsis-induced liver damage (165). Further research endeavors have enhanced therapeutic efficacy by combining RSV with silver nanoparticles, significantly decreasing inflammation markers, NF-κB activation, procalcitonin, prothrombin, and VEGF levels, with a concurrent elevation in SIRT1 levels. This suggests the scheme’s effectiveness in mitigating liver damage induced by sepsis through the further promotion of SIRT1 functionality (47).

RSV’s antioxidative and anti-nitrative properties may also impart protective effects against septic lung damage. Zhang et al. observed reductions in MDA and H2O2 levels, enhancements in the GSH/GSSG ratio, CAT, and SOD activities, suppression of iNOS expression, and NO production, along with significantly diminished formation of peroxynitrite in lung tissues of LPS model mice (166). Mechanistically, Wang and colleagues explored RSV’s potential in safeguarding against sepsis-induced acute lung injury via the PI3K/NRF2/HO-1 signaling pathway. RSV administration in rat CLP models significantly modulated bronchoalveolar lavage fluid levels of MIP-2, IL-18, and IL-10, effectively reducing wet-to-dry ratio (W/D), lung weight ratio, lung injury scores, along with MDA and 8-OHdG levels. Additionally, lung tissues exhibited increased HO-1 mRNA expression with upregulated HO-1 and Nrf-2 protein expressions, and Akt phosphorylation, thereby mitigating inflammation, oxidative stress, and apoptosis, and alleviating pulmonary damage in septic rats (167).

### 4.3 Quercetin

As of now, the ClinicalTrials.gov database lists over 70 studies related to quercetin (http://www.clinicaltrials.gov). Both preclinical and clinical research indicates that quercetin may reduce the risk of cardiovascular diseases and stroke, regulate blood sugar levels, and exhibit anti-cancer properties (168). Furthermore, a substantial body of literature highlights its potential in reducing oxidative stress and playing a beneficial role in the treatment of MODS caused by sepsis (169). Sang and colleagues observed that quercetin could ameliorate lung pathological damage and oxidative injury induced by sepsis in mice. In MLE-12 cells stimulated by LPS, quercetin decreased the mRNA expression of proteins or factors associated with endoplasmic reticulum stress and protein folding while increasing SOD levels and reducing the production of ROS and MDA. This inhibits endoplasmic reticulum stress levels and improves mitochondrial function. Transcriptome analysis revealed that quercetin upregulated the expression of SIRT1/AMPK mRNA. In this study, knocking down SIRT1 abolished the *in vitro* antioxidative effects induced by quercetin, proving that quercetin could prevent sepsis-induced lung injury by inhibiting oxidative stress-mediated endoplasmic reticulum stress and mitochondrial dysfunction through inducing the SIRT1/AMPK pathway (170).In another study, Chen et al. demonstrated that quercetin could inhibit the activation of the NLRP3 inflammasome by preventing the nuclear accumulation of PKM2 in dimeric form and increasing SIRT1 levels, thereby providing protection against septic lung injury. These findings suggest promising therapeutic strategies for septic lung injury (93) (88). As a regulator of autophagy in various forms of acute kidney injury (AKI), p53 acetylation exacerbating endothelial barrier function dysfunction induced by LPS, contributing to septic kidney damage. The activation of SIRT1 induced by resveratrol/quercetin led to the deacetylation of p53, promoting autophagy in renal tubular epithelial cells and alleviating acute kidney injury caused by sepsis. Thus, these substances might offer therapeutic effects on septic kidney injury through the p53 factor (171).

### 4.4 Tubeimuside I

Tubeimoside I (TBM) is a triterpenoid saponin purified from traditional Chinese medicine, *Bolbostemma paniculatum*. Endothelial dysfunction plays a crucial role in the progression of sepsis-induced MODS. Yang et al. found that TBM ameliorates oxidative stress by lowering the levels of NOX2 and the acetylation of SOD2, and reduces endothelial cell apoptosis by inhibiting the cleavage of caspase-3 and the Bax/Bcl-2 ratio. In models of sepsis-induced endothelial dysfunction, the expression of SIRT3 is significantly reduced, whereas TBM treatment reversed this trend, enhancing SIRT3 expression. Furthermore, pretreatment with siRNA to knock down SIRT3 considerably negated the cytoprotective effects of TBM, highlighting the pivotal role of SIRT3 in TBM’s endothelial protective mechanisms (119). Similarly, TBM, via SIRT3 modulation, also mitigates sepsis-associated myocardial damage. Under septic conditions, TBM markedly improves cardiac function, reduces inflammatory cytokine levels, and diminishes oxidative stress and apoptosis. Additionally, it reverses the trend of reduced SIRT3 expression in the heart under septic conditions, leading to an increase in its levels. Pretreatment with a specific SIRT3 inhibitor virtually nullifies the cardioprotective effects of TBM. *In vitro* experiments demonstrate that TBM shields H9c2 cells from LPS-induced damage, and the knockdown of SIRT3 diminishes this protective effect (172).

### 4.5 NAD+ boosting molecules

SIRTs are enzymes that depend on NAD+. Numerous studies have reported a decrease in NAD+ levels in various organs such as the liver, lungs, and heart during sepsis. Nicotinamide mononucleotide (NMN) and Nicotinamide ribose (NR), as the precursors of NAD+, have shown the ability to reverse the decreased NAD+ levels caused by sepsis. NMN enhances the bactericidal activity of neutrophils and macrophages, limits excessive inflammation in sepsis, and protects endothelial cell function. NMN plays a distinct organ-protective role in sepsis (76), Zhao et al. also reached similar conclusions in their experiment using NR to treat septic mouse (67). However, the organ-protective effects of NMN and NR are often counteracted by the use of SIRT1 inhibitors or SIRT1 knockout treatments. *In vitro*, Li et al. found that NMN reversed the weakened NAD+/SIRT1/PGC-1α axis in sepsis rats and improved hippocampal apoptosis, inflammation, oxidative stress. These NMN benefits above mentioned were negated by EX-527 (173). In LPS-induced AKI, NAD+ level significantly decreases and is negatively correlated with renal dysfunction. Restoring NAD+ with NMN can significantly improve LPS-induced oxidative stress and cell apoptosis, and alleviate kidney injury. Inhibition of SIRT1 weakens the protective effect of NAD+ and exacerbates AKI (150). Predictably, when exposed to LPS, EX527 counteracted the inhibitory effects of NR on HMGB1 release in macrophages, as well as on ROS and apoptosis in endothelial cells (174). These findings indicate that NMN and NR exert their effects through the NAD+/SIRT1 pathway. Regrettably, researchers have yet to explore the combination of NAD+ boosting molecules like NMN/NR with SIRTs activators for the treatment of sepsis. Whether the combination proves more beneficial in the treatment of sepsis warrants further investigation, and we eagerly await further research in this area!

### 4.6 Ginsenoside (Rg)

Ginseng remains one of the most frequently utilized herbal medicines globally, with Ginsenoside (Rg), particularly Rg1, being its primary bioactive constituent. Previous studies have highlighted Rg1’s anti-inflammatory and anti-apoptotic properties, which afford a protective effect against MODS in sepsis (175–177). Wang et al. studied inflammatory markers, oxidative stress, and pulmonary histopathology in a CLP mouse model, finding that Rg1 treatment improved lung tissue damage. It notably reduced apoptosis in LPS-induced A549 cells and lessened oxidative stress damage. Rg1 significantly decreased pro-inflammatory cytokines (TNF-α, IL-6) in A549 cells and mouse lung tissues and reduced ER stress by upregulating SIRT1 and lowering marker protein expressions (178). Additional compounds that modulate sepsis-induced multi-organ damage through the Sirtuin family are exemplified in **Table 3**.

**Table 3:** Drugs targeting SIRTs in sepsis-induced multiple organ damage.

Drugs or Compounds	SIRTs related	Organ	Reference
Melatonin	SIRT1	Brain	(154, 155)
		Heart	(156, 157)
		Liver	(158)
		Kidney	(159)
	SIRT3	Lung	(117)
		Small-Intestine	(160)
Resveratrol	SIRT1	Microcirculation	(69)
		Heart	(164)
		Liver	(165)
		Lung	(166, 167)
Quercetin	SIRT1	Lung	(93, 170)
		Kidney	(171)
TBM	SIRT3	Vascular endothelium	(119)
		Heart	(172)
Gensenoside	SIRT1	Lung	(178)
NMN	SIRT1	Brain	(173)
Amarogentin (AMA)	SIRT1	Brain	(179)
Pterostilbene	SIRT1	Liver	(180)
Emodin	SIRT1	Lung	(181)
Tanshinone IIA	SIRT1	Lung	(182)
Butein	SIRT1	Brain	(183)
	SIRT3	Heart	(184)
Rhein	SIRT1	Lung	(185)
Fgr Kinase Inhibitor	SIRT1	Brain	(114)
Polydatin	SIRT6	Heart	(186)
2,4,5-Trisubstituted Pyrimidine	SIRT5	Kidney	(187)
Caprolactone	SIRT1	Lung	(188)
Paeoniflorin	SIRT1	Liver	(189)
Trimetazidine	SIRT1	Heart	(190)
Resorcinol D1	SIRT1	Lung	(191)
Ganoderma lucidum polysaccharides	SIRT1	Heart	(192)
Eprosartan	SIRT1	Kidney	(193)
Salidroside	SIRT1	Lung	(194)
Tetrahydrochitosan	SIRT1	Kidney	(195)
Rutin	SIRT1	Kidney	(196)
Irisin	SIRT1	Kidney	(197)

## 5 Conclusion

SIRTs as critical proteins that regulate cell survival, metabolism, and stress responses, plays a significant role in the inflammation, energy metabolism dysregulation, and oxidative stress induced by sepsis. In the complex pathological process of sepsis, excessive activation of immune cells leads to the release of a profusion of inflammatory mediators, which further perpetuates the inflammatory response, causing MODS. Through their deacetylase activity, SIRTs regulates the NF-κB signaling pathway and key factors responsible for orchestrating intracellular antioxidant defenses and metabolic balance, thereby playing a pivotal role in modulating inflammation, protecting cells from oxidative stress damage, and maintaining energy homeostasis. SIRT1 and SIRT3 are particularly noteworthy because of their strong potential in anti-inflammatory, immune regulation, and mitochondrial function protection. SIRT1 reduces the expression of inflammatory cytokines by directly deacetylating the NF-κB inhibitory subunit, while SIRT3 mainly alleviates oxidative stress-induced damage by maintaining mitochondrial function and enhancing the activity of antioxidative enzymes, improving sepsis-induced MODS.

In recent years, a substantial amount of research has focused on exploring drugs and compounds that activate or enhance the function of SIRTs to mitigate sepsis and related MODS. These compounds, including melatonin, resveratrol and quercetin, have demonstrated protective effects against sepsis-induced multi-organ damage in animal models, primarily by modulating immune cell function, suppressing the inflammatory response, improving energy metabolism efficiency, and reducing oxidative stress, showcasing the feasibility of leveraging the SIRTs pathway for therapeutic efficacy.

Despite the encouraging prospects provided by current research on the application of SIRTs in the treatment of sepsis, the translation of these findings into clinical applications faces challenges. Issues such as drug selectivity, timing of treatment, dose optimization, and potential side effects require further investigation and exploration. Moreover, the interaction among members of the Sirtuin family and their specific impacts on different organ functions demand a deeper understanding.

Overall, the role of SIRTs in regulating immune responses, oxidative stress, and energy metabolism dysregulation in sepsis makes it a promising new target for treating sepsis and its associated MODS. Future work will focus on deepening the understanding of SIRTs’ function and regulatory mechanisms, as well as developing and testing more effective SIRT activators or inhibitors to improve the treatment outcomes and prognosis for sepsis patients. Concurrently, preclinical studies should advance to facilitate the clinical translation of these compounds’ therapeutic effects.

## Author contributions

JY: Writing – original draft, Writing – review & editing. YL: Writing – original draft, Writing – review & editing. WC: Writing – original draft, Writing – review & editing.
